# Exploring antibiotic resistance mechanisms in *Mycobacterium abscessus* for enhanced therapeutic approaches

**DOI:** 10.3389/fmicb.2024.1331508

**Published:** 2024-02-06

**Authors:** Thanh Quang Nguyen, Bo Eun Heo, Seunghyeon Jeon, Anwesha Ash, Heehyun Lee, Cheol Moon, Jichan Jang

**Affiliations:** ^1^Division of Life Science, Department of Bio & Medical Big Data (BK21 Four Program), Research Institute of Life Science, Gyeongsang National University, Jinju, Republic of Korea; ^2^Department of Clinical Laboratory Science, Semyung University, Jecheon, Republic of Korea

**Keywords:** *Mycobacterium abscessus*, mycobacterium drug efficacy, drug resistance, efflux pump, intrinsic-extrinsic drug resistance

## Abstract

*Mycobacterium abscessus*, a leading cause of severe lung infections in immunocompromised individuals, poses significant challenges for current therapeutic strategies due to resistance mechanisms. Therefore, understanding the intrinsic and acquired antibiotic resistance of *M. abscessus* is crucial for effective treatment. This review highlights the mechanisms employed by *M. abscessus* to sustain antibiotic resistance, encompassing not only conventional drugs but also newly discovered drug candidates. This comprehensive analysis aims to identify novel entities capable of overcoming the notorious resistance exhibited by *M. abscessus*, providing insights for the development of more effective therapeutic interventions.

## 1 Introduction

Non-tuberculous mycobacteria (referred to as NTMs hereafter) have emerged as a significant public health concern, with steadily increasing morbidity and mortality rates worldwide, eventually surpassing those of tuberculosis (Howard et al., 2006; Wassilew et al., 2016). NTM infections are opportunistic diseases primarily affecting individuals with compromised immune systems, such as patients with cystic fibrosis (*CF*), chronic obstructive pulmonary disease, renal failure, transplant recipients with chronic corticosteroid use, TNF-α, and leukemia (Faria et al., 2015). While NTM infections most commonly occur in the lungs, they can also develop in other organs. Importantly, NTM infections are rarely contagious, signifying that they do not spread from person to person, distinguishing them from other types of respiratory infections (Swenson et al., 2018; Lipman et al., 2021).

*Mycobacterium abscessus* (referred to as *Mab* hereafter) is the second most significant pathogen in NTM-induced pulmonary disease, and it is increasingly emerging as the most prominent and concerning pathogen in hospitals and *CF* centers worldwide (Degiacomi et al., 2019). *Mab* was firstly isolated in 1952 by Moore and Frerichs from a 63-year-old woman’s knee abscess and it was classified as *Mycobacterium chelonae* subsp. *abscessus* (MOORE and FRERICHS Moore and Frerichs, 1953; Victoria et al., 2021). However, *Mab* was recognized as an independent species from *M. chelonae* based on DNA hybridization and two new species *Mycobacterium massiliense* and *Mycobacterium bolletii* were described as novel and closely related to *Mab* based on the *rpoB* gene sequence (Lee et al., 2015; Lopeman et al., 2019; Victoria et al., 2021). However, since all these thee species share more than 70% relatedness based on DNA–DNA hybridization, *M. massiliense*, *M. bolletii*, and *M. abscessus* were presented as subspecies such as *Mab* subsp. *abscessus*, *Mab* subsp. *bolletti*, and *Mab* subsp. *massiliense* (hereafter referred to as *M. abscessus*, *M. bolletii*, and *M. massiliense*) and the combinations of the three subspecies were known as *Mab* complex (Lopeman et al., 2019; Victoria et al., 2021). The genome of *Mab* (CIP 104536 T) comprise 5,067,172-bp circular chromosome including 4,920 predicted coding sequences (CDS), an 81-kb full-length prophage and 5 IS elements, and a 23-kb mercury resistance plasmid almost identical to pMM23 from *Mycobacterium marinum* (Ripoll et al., 2009). *Mab* complex is responsible for 2.6–13.0% of all NTM pulmonary infections (Dedrick et al., 2023). The natural habitat of *Mab* is in soil and water sources, leading to a high rate of human-pathogen contact. Furthermore, nosocomial outbreaks and the transmission of *Mab* have been continuously occurring in clinics that conduct cosmetic surgery, liposuction, mesotherapy, or intravenous infusion of cell therapy (Lee et al., 2015; Desai and Hurtado, 2018). Nosocomial outbreaks of *Mab* through *Mab* contaminated surgical materials and hospital tap water, have also been reported as well in patients without *CF* (Baker et al., 2017; Fernandes Garcia de Carvalho et al., 2018). While it was previously believed that a significant portion of *Mab* infections in *CF* patients originated from exposure to sources such as soil, household dust, or water, potentially through contact with contaminated objects (fomites) or airborne particles (aerosols) (Falkinham, 2011), recent studies indicate that individuals with *CF* can also be infected through person-to-person transmission through hospital-based (Bryant et al., 2016). Additionally, a study by Ruis et al. suggests that dominant *Mab* circulating clones initially emerged within non-*CF* populations and were later amplified and spread within the *CF* community. Consequently, individuals with *CF* might be more permissible hosts, while non-*CF* individuals play a crucial role in transmission networks, potentially facilitating long-distance spread. This conclusion was drawn from an evolutionary phylogenetic analysis employing whole-genome sequences of clinical isolates from 1,178 *CF* and non-*CF* individuals across five continents (Ruis et al., 2021).

For the aspect of *Mab* diagnosis, there is frequent misdiagnosis of *Mab* as *M. tuberculosis* (referred to as *Mtb*), primarily due to the visual similarities observed in sputum samples under microscopic analysis (Wu et al., 2018). These circumstances not only lead to incorrect treatments but also have significant consequences, including the underestimation of NTM incidence and the inefficient allocation of budgetary resources dedicated to combating the disease. Moreover, it’s crucial to recognize that monotherapies alone are insufficient to fully eradicate the microbiological infection. According to the latest 2020 ATS/ERS/ESCMID/IDSA clinical practice guidelines, the treatment for *Mab* pulmonary disease is categorized based on macrolide susceptibility. For macrolide-susceptible cases, the guidelines recommend an initial phase with 1–2 parenteral drugs (amikacin; AMK, imipenem; IMP, cefoxitin; CFX, and tigecycline; TGC) and two oral drugs (azithromycin; AZM, clofazimine; CFZ, and linezolid; LZ), along with inhaled AMK. In the case of macrolide-resistant organisms, the recommendations include an initial phase with 2–3 parenteral drugs (AMK, IMP, CFX, and TGC) and 2–3 oral drugs (AZM, CFZ, and LZ), supplemented with inhaled AMK. The addition of AZM is for its immunomodulatory effect, although adherence to these guidelines may have adverse effects on NTM patients (Moguillansky et al., 2023). However, *Mab* has demonstrated resistance to a broad spectrum of antibiotics, including the aforementioned treatment regimen, and patients experience multiple relapses with low cure rates, making it challenging to achieve a complete cure (Victoria et al., 2021). This discouraging success rate primarily stems from the rapid development of drug resistance, which can be attributed to both intrinsic and acquired multidrug resistance to antibiotics. Notably, even first-line anti-TB medications, such as isoniazid (INH) and rifampicin (RFP), lack efficacy against *Mab* (Zheng et al., 2023). As a result, the majority of *Mab* treatment protocols involve extended multi-antibiotic regimens that can last up to 24 months (Wu et al., 2018; Ratnatunga et al., 2020). However, the effectiveness of these treatments remains limited, with disease remission rates reaching only 30% (Wu et al., 2018; Ratnatunga et al., 2020). Additionally, in cases of pulmonary infections, no class of antibiotics has demonstrated the ability to achieve long-term sputum smear conversion (Wu et al., 2018; Ratnatunga et al., 2020).

The three subspecies of *Mab* shows distinct clinical outcomes (Blauwendraat et al., 2012; Harada et al., 2012; Shin et al., 2013; Jeong et al., 2017; Abate et al., 2019). Firstly, *M. abscessus* exhibits resistance to macrolides such as AZM and clarithromycin (CLR) due to an adaptive resistance mechanism involving the inducible erythromycin ribosomal methyltransferase, *erm(41)* (Stout and Floto, 2012; Rubio et al., 2015; Christianson et al., 2016; Abate et al., 2019). Consequently, the use of macrolides in treating *M. abscessus* infections should be approached with great caution (Maurer et al., 2014). Secondly, *M. massiliense* is the most recent subspecies within this group and has a broader geographical distribution compared to the other subspecies. Notably, this subspecies tends to yield more favorable clinical outcomes than the other two, primarily because it lacks the functional *erm* gene. Lastly, *M. bolletii* represents the rarest among the three subspecies and is also resistant to CLR.

However, our understanding of the intrinsic or acquired antibiotic resistance of *Mab* remains limited. Therefore, alongside the ongoing efforts to discover novel alternative compounds for *Mab* treatment, it is crucial to elucidate the resistance mechanisms employed by *Mab* against existing antibiotics. This endeavor not only aids in enhancing the effectiveness of current antibiotics to overcome these resistance barriers but also provides valuable insights for the development of new compounds. This review aims to offer a comprehensive overview of the current knowledge of antibiotic resistance mechanisms in *Mab*, with the goal of clarifying the molecular components contributing to its significant resistance to chemotherapy and facilitating the development of a drug pipeline for *Mab*.

### 1.1 Drug discovery and limitations

The quantity of initiatives in antimicrobial drug development has significantly diminished since the remarkable era of antibiotic discovery, and several factors contribute to this decline. Firstly, the increasing prevalence of drug-resistant bacteria limits the effectiveness of new antibiotics, making it challenging to recoup investments in antibiotic development. Secondly, antibiotics are typically prescribed for short durations, in contrast to drugs for chronic conditions like hypertension or diabetes, which may render antibiotics less financially appealing to pharmaceutical companies. Thirdly, novel effective drugs are often preserved as last-resort treatments for highly-resistant bacterial infections. The goal is to mitigate the development of further resistance by limiting their widespread use. Overuse or misuse of these potent drugs can accelerate the emergence of resistant strains, making them ineffective sooner. Overexposure can diminish their efficacy over time, making it crucial to reserve them for cases where no other options are viable (Baker et al., 2017). Lastly, the discovery of new antibiotics presents scientific challenges (Ventola, 2015; Quang and Jang, 2021). Finding compounds that are both effective against bacteria and safe for humans is a complex process, and success is not guaranteed (Chopra et al., 2011). The identification and development of innovative and potent medications for combating NTMs are of paramount importance in the medical field. In comparison to the tuberculosis drug pipeline, which features a significant number of compounds undergoing clinical trials, the NTMs drug pipeline lags considerably (Wu et al., 2018). Notably, there is a critical need for the development of new treatments targeting *Mab* as there are currently no antibiotics approved by the Food and Drug Administration (FDA) for *Mab* infection.

Two primary strategies exist to bolster the development of effective *Mab*-targeting medicines. The first strategy follows the conventional drug development process, which encompasses the identification of novel chemical compounds. This process commences with drug screening using chemical and natural product libraries, progressing through hit identification, lead optimization, target identification, comprehensive preclinical investigations, and ultimately clinical trials (Egorova et al., 2021). Various screening methods have been employed in this pursuit for *Mab* drug discovery, including reporter-based assays, resazurin-based microplate assays, and image-based phenotypic screens (Gupta et al., 2017; Jeong et al., 2018; Richter et al., 2018; Kim et al., 2019; Malin et al., 2019; Hanh et al., 2020a,b). Nevertheless, despite these efforts, promising new chemical leads ready for clinical trials and market release remain scarce (Hanh et al., 2020a). This challenge may be attributed to the intrinsic drug-resistant mechanisms of *Mab*, resulting in a low hit rate for compounds targeting this bacterium (Malin et al., 2019). It’s noteworthy that the hit rate achieved in *Mab* screens is significantly lower than what is typically observed in screens targeting *Mtb* (Malin et al., 2019; Hanh et al., 2020a). Moreover, recent *Mab* drug screens have relied on conventional libraries composed of known antimycobacterial or antibacterial agents (Malin et al., 2019), diminishing the likelihood of identifying novel compounds targeting new mechanisms of action. Hence, there is an urgent need to develop new libraries with expanded chemical diversity to discover unique compounds. Additionally, reliable cell-based or *in vivo* assessment/screening methods that accurately mimic the host environment infected with *Mab* are imperative to advance our understanding and discovery of effective treatments for *Mab*-induced human infections (Carvalho et al., 2011; Bernut et al., 2014, 2015). The second strategy involves repurposing or repositioning existing medications for novel therapeutic indications. Most contemporary antibiotics and potential prospects against *Mab* have origins in the repurposing of pre-existing drugs or the cross-testing of compounds with activity and various mechanism of action against *Mtb* (Egorova et al., 2021). This method is particularly intriguing in the field of antibacterials, as the rapid evolution of resistance often outpaces the pace of medication development (Egorova et al., 2021). Repurposing previously approved pharmaceuticals can expedite the development process and reduce expenses (Egorova et al., 2021). Regrettably, the *Mab* drug pipeline remains underpopulated (Ganapathy and Dick, 2022). Currently, there are four recruiting, four completed, one terminated, and two non-recruiting clinical trials evaluating drug efficacy in *Mab* infection (NIH ClinicalTrials, 2023). However, these clinical trials have primarily utilized existing antibiotics through various drug delivery methods, including inhalation, novel drug encapsulation using biocompatible liposomes, and the exploration of new drug combinations (Quang and Jang, 2021). The primary reason for the limited success in anti-*Mab* drug discovery is the remarkable intrinsic resistance capabilities of *Mab* and its rapid acquired resistance against currently available active drugs (Wu et al., 2018).

## 2 Mechanisms of *Mab* resistance to current antibiotics

**Figure 1:**
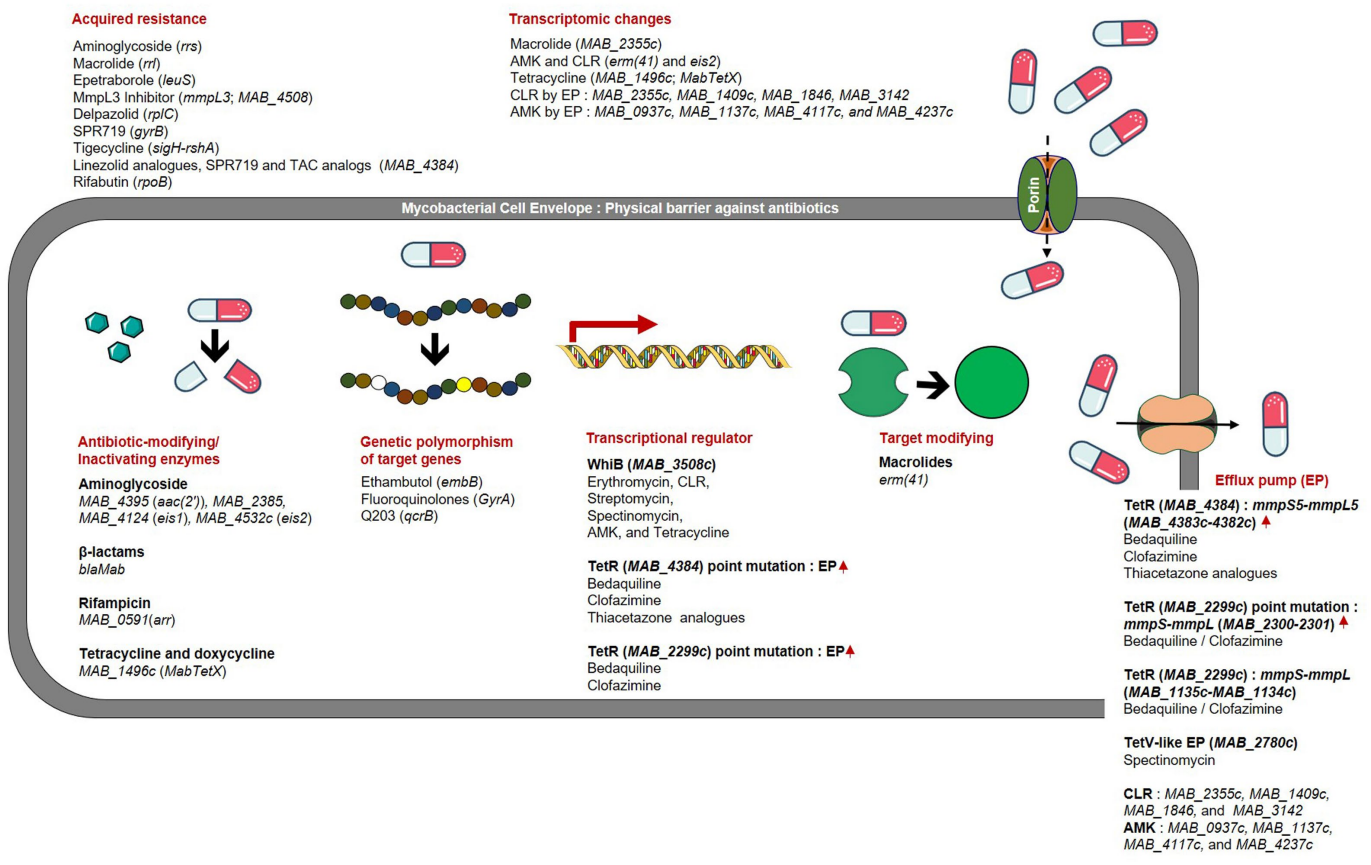
Drug resistance mechanisms and related genes in *Mab.*

Inherent drug resistance in NTMs is responsible for their limited susceptibility to a wide range of medicines and chemicals (Wu et al., 2018). This inherent resistance in *Mab* and other mycobacterial species can be attributed to several factors, including the presence of a waxy impermeable cell wall that acts as both a physical (size exclusion) and a chemical (hydrophobic) barrier, drug export systems, enzymes capable of modifying drugs or target enzymes, and genetic polymorphisms in target genes (Nessar et al., 2012) (Figure 1). In addition to harboring numerous intrinsic resistance mechanisms, *Mab* possesses the ability to acquire novel resistance through genetic changes that can be passed down to subsequent generations. Acquired resistance is not linked to genes introduced by transmissible genetic elements like plasmids and transposons (Martin et al., 1990). Instead, resistance arises due to spontaneous mutations occurring at specific genes in response to the presence of antibiotics following extended courses of treatment (Johansen et al., 2020b). This allows bacteria to undergo genetic changes in the target gene or other associated genes, resulting in the acquisition of significant levels of resistance, rendering the medicine ineffective. However, species or subspecies may exhibit variations in their antibiotic resistance phenotype and genotype, emphasizing the need for research on accurately identified strains (Nessar et al., 2012). In this section, we focus on intrinsic and acquired resistance to essential drugs and new drug candidates that have demonstrated efficacy against *Mab*.

### 2.1 Mycobacterial cell envelope

**Figure 2:**
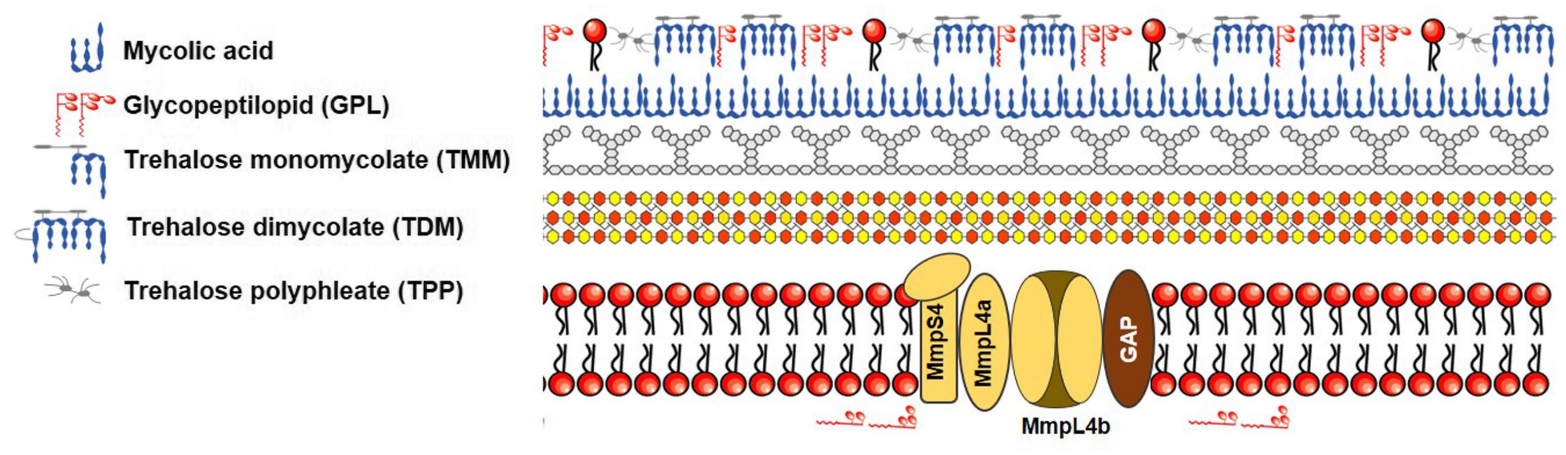
Schematic representation of mycobacterial cell envelope based on the figure of Gutiérrez et al.

Mycobacteria’s cell wall is primarily composed of lipids, specifically mycolic acids, constituting a significant portion (up to 60%) of the bacteria’s overall dry mass (Brennan and Nikaido, 1995). This cell wall features a waxy composition that serves as a physical barrier (Nessar et al., 2012), rendering mycobacteria less permeable than the outer membranes of gram-negative bacteria. In more detail, the mycobacterial envelope consists of three distinct layers: a typical plasma membrane, a complex cell wall, and an outer layer. The cell wall, notably, comprises a thick peptidoglycan layer covalently linked to arabinogalactan, which is esterified by mycolic acids, forming the inner leaflet of the mycomembrane (Figure 2). This unique structure inherently makes mycobacteria resistant to many antimicrobials. Hydrophilic drugs penetrate the mycobacterial cell wall slowly due to the inefficiency of mycobacterial porin in allowing antibiotic permeation, resulting in low antibiotic concentrations within the bacteria. The dense mycobacterial cell wall not only shields the bacterium from stressors but also poses challenges in nutrient uptake from the environment. To address this, mycobacteria often produce porins, proteins that create limited pathways for nutrient absorption. The expression of these porins is closely tied to the growth rate of NTMs, and they provide a conduit for certain antimicrobial agents to enter the mycobacterial cell (Saxena et al., 2021). Lipophilic agents may be hindered by the lipid bilayer, which has unusually low fluidity and thickness (Jarlier and Nikaido, 1994; Gutiérrez et al., 2018). Intriguingly, *Mycobacterium chelonae*, a species closely related to *Mab* due to its nearly identical biochemical features, has a cell envelope that is about 10–20 times less permeable than that of *Mtb* (Jarlier and Nikaido, 1994). Similar to *Mtb*, *Mab* possesses a mycobacterial cell wall with low permeability, which contributes to its drug resistance. Notably, like *Mtb*, *Mab* is thought to regulate cell wall structure and homeostasis through lipoprotein glycosylation. For example, the absence of protein-O-mannosyltransferase Pmt (*MAB_1122c*) in *Mab* leads to increased cell wall permeability and greater susceptibility to antibiotics such as RFP (Ganapathy et al., 2019). Furthermore, glycosylation of lipoproteins limits cell wall permeability to antibiotics like β-lactam agents that inhibit peptidoglycan synthesis. In β-lactam drug resistance, mycobacterial porins also play a role by facilitating the transport of small hydrophilic drugs across the membrane. Once antibiotics are internalized, they can reach their target in the cytoplasm and activate potential internal drug resistance mechanisms, collectively known as the “intrinsic resistome.” This resistome includes efflux pumps, antibiotic-modifying/inactivating enzymes, target-modifying enzymes, and genes conferring metal resistance (Nguyen and Thompson, 2006; Nessar et al., 2012).

Remarkably, unlike *Mtb*, *Mab* exhibits two distinct colony morphotypes: smooth non-cording (S) and rough cording morphotype (R). These differences in morphotypes depend on the presence or absence of cell surface-associated glycopeptidolipids (GPL), respectively (Howard et al., 2006). This distinctive property, associated with GPL status, affects sliding motility, biofilm formation, and drug susceptibility. For example, S morphotype strains that contain GPL, such as *M. abscessus* and *M. bolletii*, facilitate sliding across the surface and biofilm formation. Indeed, the *Mab* growing inside biofilms become tolerant to antibiotics due to physical barrier that can prevent the intracellular penetration of compounds. In fact, *in vitro* biofilm models of *Mab* have been exhibited to have decreased susceptibility to several first-line antibiotics, such as cefoxitin, amikacin, and clarithromycin (Greendyke and Byrd, 2008; Marrakchi et al., 2014). Conversely, R morphotype strains exhibit aggregation and cording. Recent studies suggest that *Mab* R strains are capable of growing in biofilm-like structures, which, similar to S biofilms, show greater tolerance than planktonic cultures to acidic pH, hydrogen peroxide, and treatment with antibiotics like AMK, AZM, and β-lactams (Gutiérrez et al., 2018; Story-Roller et al., 2018; Daher et al., 2022; López-Roa et al., 2022). Furthermore, biofilms formed by R colony types display higher mechanical resistance compared to those formed by S colony types (López-Roa et al., 2022). Biofilms also inhibit oxygen and nutrients from entering the cell, which causes a reduction *Mab* metabolism and, consequently, an increased tolerance to a harsh environment such as antibiotics treatment. Therefore, compounds that specifically target biofilm formation during antibiotic therapy are a new therapeutic strategy for clearance of *Mab.*

In invasive infections causing pulmonary colonization, *Mab* R strains are accountable for producing higher levels of trehalose dimycolate, consequently leading to the formation of massive bacterial cords. With the rough variant, the entire phagosome quickly merges with the lysosome, inducing phagosomal acidification and activating apoptosis and autophagy (Roux et al., 2016; López-Roa et al., 2022). This robust apoptosis-driven cell-death activity facilitates the extracellular replication of the R variant through rapid cord formation, preventing the engulfment of bacilli by neutrophils and macrophages. This process leads to abscess formation, tissue destruction, and acute infection (Jönsson et al., 2013; Bernut et al., 2016; López-Roa et al., 2022).

### 2.2 Antibiotic-modifying/inactivating enzymes and acquired drug resistance

#### 2.2.1 Aminoglycosides (AGs)

*Mab* produces enzymes capable of modifying antibiotics by cleaving, altering their structure, and adding or removing chemical groups (Luthra et al., 2018). These modifications can render antibiotics ineffective by either preventing their binding to their target or increasing their susceptibility to hydrolysis by the bacteria (Luthra et al., 2018). The efficacy of antibiotics was restored when these modifying genes were knocked down (Nessar et al., 2012). AG antibiotics are composed of amino carbohydrates linked by glycoside bonds (Mingeot-Leclercq et al., 1999). Among antibacterial drugs, AMK has shown the most efficacy against *Mab* (Tsai et al., 2015). AGs can diffuse through porins and interact with 30S ribosomes (e.g., streptomycin), 50S ribosomes (others), or both 30S and 50S ribosomes (Chulluncuy et al., 2016). This interaction prevents the initiation of protein synthesis, the continuation of translation, or the incorporation of incorrect proteins (Bhattacharjee, 2016). Unfortunately, *Mab* has developed resistance to aminoglycosides. *Mab’s* genome annotation suggests the presence of various AG-modifying enzymes, including AG phosphotransferases, AG nucleotidyltransferases, and AG acetyltransferases (AACs). Among these, AG AAC (2′-N-acetyltransferase) and AG phosphotransferases render AG antibiotics inactive by transferring acetyl or phosphate residues to crucial positions within the antibiotic (Nessar et al., 2012) (Supplementary Figures S1, S2). *Mab*’s genome analysis and AG drug susceptibility testing indicate the presence of several putative AACs, which acetylate aminoglycosides with a 2′ amino group, such as gentamicin, tobramycin, and KM. ORF *MAB_4395* is annotated as a putative AG 2′-N-acetyltransferase [*aac(2′)*], and *aac(2′)* deletion mutants increased *Mab*’s susceptibility to KM-B, tobramycin, dibekacin, and gentamicin C (Rominski et al., 2017b). Furthermore, *MAB_2385*, which serves as the main determinant of resistance to the first-discovered aminoglycoside, streptomycin, functions as a 3″-O-phosphotransferase. Deletion of *MAB_2385* in *Mab* increases susceptibility to streptomycin, while introducing *MAB_2385* in *M. smegmatis* (*Msm*) confers resistance to streptomycin (Dal Molin et al., 2017). In addition to *MAB_4395* and *MAB_2385*, *Mab* possesses additional AG-modifying enzymes named Eis (enhanced intracellular survival), including *MAB_4124* (also known as *eis1*; sharing 33% identity with *Mtb Rv2416c*) and *MAB_4532c* (*eis2*), which are involved in AG resistance. *MAB_4532c* significantly enhances *Mab*’s intracellular survival and has been shown to modify KM, hygromycin, and AMK *in vitro* (Ung et al., 2019). Deletion of *MAB_4532c* strains increased *Mab*’s susceptibility to AGs and capreomycin (Rominski et al., 2017b; Johansen et al., 2020b). Furthermore, MAB_4532c is responsible for the lack of bactericidal activity of AMK *in vitro* and affected AMK activity *in vivo* (Lorè et al., 2022; Selchow et al., 2022).

Clinically acquired pan-AG resistance is linked to mutations in ribosomal RNA genes, specifically *rrs*, which encode the 16S rRNA molecule as acquired resistance (Rominski et al., 2017b). The prolonged use of AGs can lead to genetic modifications in *rrs* (Wallace et al., 1996; Prammananan et al., 1998; Maurer et al., 2012). It’s worth noting that the substitution of adenine with guanine at position 1408 (A1408G) in *rrs* significantly increases resistance to KM, AMK, and tobramycin (Nessar et al., 2011). Recently, two novel *rrs* mutations, C1496T and T1498A, were also identified from *Mab*-pulmonary disease patients (Young et al., 2021). Additionally, mutations at locations T1406A, C1409T, and G1491T in *rrs* could potentially confer a high level of resistance to KM, AMK, and gentamicin (Nessar et al., 2011).

Apramycin (AP; also known as Nebramycin II) is presently authorized by the Veterinary Medicines Directorate in the UK for use in pigs, cattle, rabbits, and chickens. It is available either as (i) a premix for medicated feedstuff (200 g/kg, 100,000 IU/g, 100 g/kg) or (ii) a soluble powder for oral solution, with a concentration of 10% or less (Moore et al., 2018). AP has a distinctive AG structure and demonstrates potent activity against *Mab* (Selchow et al., 2022). Furthermore, it displays minimal cross-resistance to other aminoglycosides and exhibits favorable therapeutic lung exposure and a low toxicity profile (Matt et al., 2012; Ishikawa et al., 2019; Juhas et al., 2019; Becker et al., 2021; Selchow et al., 2022). Recently, Selchow et al., reported that AP is not modified by Eis2 or Aac(2′) and is not affected by the multidrug resistance regulator WhiB7. This favorable feature of apramycin is reflected in a mouse model of pulmonary *Mab* infection, which demonstrates superior activity, compared with amikacin (Selchow et al., 2022). At present, there are no established antibiotic breakpoints for AP against *Mab* provided by organizations like EUCAST (The European Committee on Antimicrobial Susceptibility Testing) or CLSI (Clinical & Laboratory Standards Institute). Pharmacokinetic/pharmacodynamics studies are essential to address this gap for AP in treating chronic *Mab* infections in humans. This evaluation process holds significant importance for approval by the FDA and the European Medicines Agency (EMA) (Moore et al., 2018). Albeit, AP is currently promising agent as Mab treatment option together with other candidates such as RFB and omadacycline (OMC).

#### 2.2.2 β-Lactam

Beta-lactams are a class of antibiotics characterized by a four-atom beta-lactam ring (De Rosa et al., 2021). They are among the most widely prescribed antibiotics due to their broad spectrum of activity against bacteria (De Rosa et al., 2021). Beta-lactam antibiotics are bactericidal because they inhibit the cross-linking or transpeptidation of the peptidoglycan layer in bacterial cell walls by covalently binding to penicillin-binding proteins (PBPs). Bacterial enzymes that hydrolyze peptidoglycan cross-links continue to function even when PBPs are inactivated by beta-lactam antibiotics, leading to further degradation of the cell wall. The buildup of peptidoglycan precursors activates cell wall hydrolases, ultimately causing the cells to burst (Bush and Bradford, 2016). To counteract the effects of beta-lactam antibiotics, *Mab* possesses a beta-lactamase-encoding gene, namely *blaMab*, which serves to break down beta-lactam antibiotics, rendering them ineffective (Dubée et al., 2015a) (Supplementary Figure S3). BlaMab efficiently degrades multiple β-lactams, surpassing the activity of BlaC, the principal β-lactamase of *Mtb*. Deletion of *blaMab* in a recombinant *Mab* strain increased its susceptibility to β-lactams, making it responsive to antibiotics like amoxicillin and ceftaroline (Lefebvre et al., 2016). Moreover, BlaMab exhibits reduced susceptibility to common β-lactamase inhibitors, such as clavulanate, tazobactam, and sulbactam, unlike inhibitors of BlaC in *Mtb* (Soroka et al., 2017). Additionally, *M. massiliense* harbors an additional β-lactamase, BlaMmas (Ramírez et al., 2017).

However, recent combination studies have shown that non-β-lactam-based β-lactamase inhibitors known as diazabicyclooctane (DBO) inhibitors, including avibactam, effectively inhibit BlaMab. This inhibition leads to a reduction in the minimum inhibitory concentration (MIC) of carbapenems and cephalosporins against *Mab* to clinically achievable levels. Currently, avibactam is exclusively marketed in combination with the cephalosporin ceftazidime under the name Avycaz in the United States (Dubée et al., 2015a; Lefebvre et al., 2017; Meir et al., 2018; Le Run et al., 2019; Kaushik et al., 2019b; Dousa et al., 2022). Furthermore, Dousa et al. have demonstrated the effectiveness of two new non-β-lactam-based β-lactamase DBO inhibitors, relebactam and vaborbactam, when evaluated in combination with various commercially available β-lactams against clinical isolates of *Mab*. In their study, both relebactam and vaborbactam significantly enhanced the anti-*Mab* activity of several carbapenems (IMP and meropenem) and cephalosporins (cefepime, ceftaroline, and cefuroxime) (Dubée et al., 2015b). Currently, the IMP-relebactam combination is undergoing phase III trials, and the meropenem-vaborbactam combination is already available in the market. This established and effective combination opens the door to using potent β-lactams for the treatment of *Mab* infections (Kaushik et al., 2019b). Furthermore, more recent DBO class β-lactamase inhibitors, including nacubactam, zidebactam, and durlobactam, have been suggested as potent β-lactamase inhibitors that can restore susceptibility to β-lactams against *Mab in vitro* (Dubée et al., 2015a; Lefebvre et al., 2017; Meir et al., 2018; Le Run et al., 2019; Kaushik et al., 2019b; Dousa et al., 2022).

#### 2.2.3 Rifampicin

Rifampicin (RFP) stands as the first-line treatment for *Mtb*, primarily due to its ability to halt transcription by binding to the beta-subunit of RNA polymerase encoded by *rpoB*. This enzyme is pivotal for bacterial transcription (Piccaro et al., 2014). This interaction prevents the bacterium from transcribing essential genetic material, ultimately leading to its demise. However, RFP is notably ineffective against *Mab*. In *Mab*, RFP’s efficacy is nullified due to the presence of the Arr gene (*MAB_0591*) (Rominski et al., 2017a). This gene produces an RFP ADP-ribosyltransferase homolog, which inactivates rifamycins by catalyzing ADP-ribosylation at position C_23_ (Rominski et al., 2017a) (Supplementary Figure S4). ADP-ribosylation confers innate rifamycin resistance in *Mab* (Ganapathy et al., 2019). Deletion of *MAB_0591* in *Mab* has proven to not only decrease the MIC for RFP but also enhance susceptibility to RFP analogs like rifaximin rifabutin (RFB) and rifapentine (Rominski et al., 2017a; Schäfle et al., 2021). For instance, the removal of this gene results in a significant increase in *Mab*’s susceptibility to RFP, rifapentine, and rifaximin, with a 100-fold reduction in RFP’s MIC (Johansen et al., 2020b; Zheng and Lupoli, 2021). Moreover, recent research has highlighted the potential of a rifamycin analogue known as RFB following its identification through drug screening. However, although being a substrate of Arr, RFB has demonstrated promising anti-*Mab* effects both *in vitro* and *in vivo*. In these studies, RFB not only inhibited *Mab* growth but also exhibited bactericidal properties against all three *Mab* subspecies. Particularly, RFB displayed comparable activity to clarithromycin (CLR) against *Mab* K21 in NOD.CB17-Prkdcscid/NCrCrl mice. These *in vitro* and *in vivo* findings suggest that RFB may enhance cure rates and shorten treatment duration for the predominantly challenging *Mab* lung disease. Hence, it should be considered a viable clinical candidate for patients with *Mab* infections (Dick et al., 2020; Johansen et al., 2020a).

Recently, 25-O-desacetyl-25-O-nicotinoylrifabutin (RFB-5 m), a new rifabutin analogue, overcomes inherent rifamycin resistance caused by Arr (Ganapathy et al., 2023b). RFB-5 m prevents enzymatic oxidation by maintaining rifabutin’s naphthoquinone core (Lan et al., 2022; Ganapathy et al., 2023b). Importantly, RFB-5 m’s unique C25 group prevents Arr *Mab* ADP-ribosylation (Lan et al., 2022; Ganapathy et al., 2023b). Compared to rifabutin, RFB-5 m is 50 times more effective against *Mab* (Lan et al., 2022; Ganapathy et al., 2023b). Moreover, RFB-5 m was observed to display bactericidal properties against the persisters of *Mab* in caseum (Lan et al., 2022). RFB-5 m also had strong enhanced potency against all members of the *Mab* complex, other clinically relevant rapidly and slowly growing NTM, all of which encode Arr and block ADP-ribosylation (Ganapathy et al., 2023b). Recently, Paulowski et al. reported that benzyl piperidine rifamycin derivative known as 5j, which possesses a morpholino substituted C3 position and a naphthoquinone core, does not undergo any modifications when exposed to pure Arr (Paulowski et al., 2022). The thermal characterization of Arr in the presence of 5j, RMP, or RFB reveals that 5j exhibits no binding affinity towards Arr (Paulowski et al., 2022) and 5j also has substantial antibiotic efficacy against *Mab* within human macrophages, and exhibits synergistic effects when combined with AMK and AZM (Paulowski et al., 2022).

Additionally, Hanh et al. recently shed light on the activity of Rifamycin O, a derivative of rifamycin resulting from the oxidation of natural rifamycin B, against *Mab*. In their study, Rifamycin O exhibited promising *in vitro* activity (MIC90 = 4.0–6.2 μM) and demonstrated comparable *in vivo* efficacy to RFB using a zebrafish (*Danio rerio*) infection model (Hanh et al., 2020b). This suggests that certain rifamycin analogs like RFB and Rifamycin O can evade *MAB_0591*-mediated rifamycin resistance mechanisms. Of note, RFB and Rifamycin O exhibit distinct chemical structures at positions C_1_ and C_4_, setting them apart from other rifamycin analogs such as RFP, rifapentine, rifamycin SV, and rifaximin. These other analogs contain hydroquinone, which can readily oxidize into RFP quinone in the presence of oxygen and divalent cations. In contrast, RFB and Rifamycin O lack hydroquinone, granting them resistance to autoxidation and thereby ensuring their efficacy against *Mab* even under oxidative conditions. Consequently, it is conjectured that the unique structural attributes at C_1_ and C_4_ of rifamycin analogs are pivotal for their effectiveness against *Mab* (Hanh et al., 2020b; Ganapathy et al., 2021b). Recently, RFB was redesigned strategically to enhance its potency against *Mab*. Modifications at the C-25 position yielded analogs over a hundred times more powerful than RFP and resistant to *Mab* ADP-ribosylation. Molecular studies highlighted additional interactions, contributing to their superior on-target effectiveness. Validated *in vitro*, these compounds effectively countered Arr-mediated resistance, displaying potent *in vivo* efficacy comparable to clarithromycin against *Mab*. The compound 5 m, exemplary candidate in aspect of antibacterial activity, excellent drug disposition, and significantly improved *in vivo* pharmacokinetic traits is ongoing investigations to unveil its *in vivo* efficacy (Lan et al., 2022).

### 2.3 Target-modifying enzymes and acquired drug resistance

#### 2.3.1 Macrolides

Macrolides represent an antibiotic class that can effectively combat a wide range of bacterial types, including staphylococci, streptococci, mycoplasma, and more. Macrolides are characterized by their structure, comprising amino sugar and/or neutral sugar components linked to a lactone ring, forming macrolides with 12-, 14-, 15-, or 16-membered rings through glycosidic connections. Their mode of action involves binding to the 50S ribosomal subunit in bacteria, which results in the inhibition of protein synthesis (Dinos, 2017). Macrolides remain the core drugs for treating *Mab* infections (Guo et al., 2021). In certain NTMs, exposure to macrolides triggers the production of specific enzymes that modify the drug’s target binding site. For example, resistance to clarithromycin (CLR), whether intrinsic or acquired, is associated with *erm(41)* and mutations in the gene *rrl* encoding a 23S peptidyl transferase in the large 23S ribosomal subunit, respectively (Nessar et al., 2012). In *M. abscessus* and *M. bolletii*, inducible macrolide resistance occurs due to a T-C polymorphism in *erm(41)* at position 28 (only isolates with T28 develop resistance) (Nash et al., 2009). Interestingly, *M. massiliense* isolate exhibits susceptibility to macrolides due to a non-functional *erm(41)* caused by a 274-bp deletion. As a result, determining subspecies and macrolide susceptibility is crucial for guiding appropriate treatment. More recently, Guo et al. identified another macrolide-resistant gene in *Mab*. The gene *MAB_2355c* exhibits ATP hydrolysis activity and contributes to macrolide resistance by protecting ribosomes (Guo et al., 2021). Expression of *MAB_2355c* mRNA is significantly upregulated after exposure to macrolides compared to other ribosome-targeting antibiotics. Deletion of *MAB_2355c* in *Mab* strains resulted in increased sensitivity to macrolides, while complemented strains exhibited reduced sensitivity to macrolides (Guo et al., 2021).

Acquired resistance to macrolides in a clinical setting often arises from spontaneous mutations at positions 2058 and 2059 on the *rrl* gene. Additionally, Vester et al. demonstrated that mutations at positions 2057 and 2,611 on *rrl* can lead to low-level resistance although these mutations are located outside the primary site of macrolide interaction (Vester and Douthwaite, 2001).

### 2.4 Transcriptional regulator WhiB gene family

*Mab* possesses a family of transcriptional regulators that may play a role in conferring drug resistance, particularly the WhiB gene family (Morris et al., 2005). WhiB7, a transcriptional activator belonging to the WhiB family of transcriptional regulators, is conserved in actinomycetes and regulates critical cellular processes, including cell division, pathogenesis, and oxidative stress responses. The presence of a helix-turn-helix motif indicates its DNA-binding function (Soliveri et al., 2000; Burian et al., 2012; Nessar et al., 2012). In *Mab*, 128 genes, including *erm(41)* and *eis2*, have been identified in the WhiB7 regulon, indicating their induction through a WhiB7-dependent mechanism. Deletion of *Mab whiB7* (*MAB_3508c*) renders the bacteria more susceptible to antibiotics, such as erythromycin, CLR, streptomycin, spectinomycin (SPC), AMK, and tetracycline, although it does not affect resistance to RFP or INH (Hurst-Hess et al., 2017). Significantly, exposing *Mab* to sub-inhibitory concentrations of CLR leads to the activation of *whiB7* gene expression (Pryjma et al., 2017). This activation subsequently results in the development of resistance to AMK and CLR due to increased expression of *erm(41)* and *eis2* genes (Pryjma et al., 2017; Johansen et al., 2020b).

### 2.5 Tetracycline

Tetracycline molecules feature a linear fused tetracyclic core to which several functional groups are attached. These compounds are known to inhibit bacterial growth by preventing the binding of charged aminoacyl-tRNA to the ribosomal A site (Chopra and Roberts, 2001). Tigecycline (TGC) became the first glycylcycline antibiotic approved by the US FDA (Stein and Craig, 2006). While tetracyclines have been one of the most successful classes of antibiotics, their widespread use has led to extensive drug resistance, necessitating their discontinuation in treating various bacterial infections (Rudra et al., 2018). Occasionally, tetracyclines become ineffective due to TetX enzymes, also known as tetracycline destructases. In the past, the limited tolerance of *Msm* and *Mtb* to tetracycline was attributed to the WhiB7-dependent TetV/Tap efflux pump. However, *Mab* exhibits a resistance level approximately 500-fold higher than that of *Msm* and *Mtb*. Recently, Rudra and colleagues revealed that this heightened resistance in *Mab* to the tetracycline class is conferred by a WhiB7-independent tetracycline-inactivating monooxygenase, *MAB_1496c* (*MabTetX*). Exposure to sublethal doses of tetracycline and doxycycline leads to a more than 200-fold induction of *MabTetX*. Conversely, an isogenic deletion strain shows high sensitivity to both antibiotics. The authors also demonstrated that *MabTetX’*s expression is suppressed by *MabTetRx*. This finding highlights the potential use of an inhibitor to potentially reinstate the effectiveness of tetracycline and doxycycline (Rudra et al., 2018). As for acquired drug resistance, mutations in the *sigH-rshA* genes, which regulate heat shock and oxidative-stress responses, have also been found to be involved in TGC resistance or decreased sensitivity in *Mab* (Ng and Ngeow, 2022). Overexpression of the *sigH* gene, resulting from the C51R mutation in *rshA*, causes resistance to or decreased susceptibility to TGC (Ng and Ngeow, 2022).

Recently, OMC, which is classified as a tetracycline antibiotic, has exhibited favorable MIC against various *Mab* species (Singh et al., 2023). The activity and efficacy of OMC against *Mab* infection have been proven in both *in vitro* and *in vivo* (Shoen et al., 2019; Watkins and Deresinski, 2019; Kaushik et al., 2019a; Brown-Elliott and Wallace, 2021; Nicklas et al., 2022). OMC, like other tetracycline derivatives via binding to the tetracycline-binding site of the bacterial 16S ribosomal RNA, inhibiting bacterial protein synthesis (Brown-Elliott and Wallace, 2021). In contrast to first-generation tetracyclines, OMC has been intentionally engineered to bypass ribosomal protection and tetracycline efflux mechanisms. In *Mab*, the production of a monooxygenase enzyme may degrade tetracyclines, such as minocycline and doxycycline, but does not affect OMC (Luthra et al., 2018; El Ghali et al., 2023). This feature, in part, revitalizes its activity against recalcitrant MAB and may serve as a deterrent against the development of resistance during treatment. For example, studies by Bax et al. revealed that, except for a 1.5% occurrence at an OMC concentration of 4 mg/L, no instances of drug resistance selection exceeded the spontaneous mutation frequency (Bax et al., 2019).

### 2.6 Efflux pumps (EPs)

Active efflux mechanisms have been identified as potential contributors to antibiotic resistance in mycobacteria. The primary role of efflux pump (EP) systems is to protect bacteria from harmful substances, maintain cellular homeostasis, and uphold physiological equilibrium by expelling toxins or metabolites into the extracellular environment (Louw et al., 2009; Remm et al., 2022). *Mab* possesses genetic sequences that encode protein constituents belonging to the major facilitator family ABC transporters and mycobacterial membrane protein large (MmpL) families (Ripoll et al., 2009), although the precise contribution of EPs to antibiotic resistance in *Mab* is not fully elucidated. ABC-type multidrug transporters utilize ATP energy to actively remove compounds from the cellular environment. MmpL transporters are multidrug EPs that play a crucial role in transporting various substrates from the periplasmic space to the extracellular environment (Kerr, 2002; Kerr et al., 2005). In further detail, the MmpL transporter family encodes proteins belonging to the resistance, nodulation, and cell division (RND) category. These proteins function as multidrug resistance pumps with the ability to transport a wide range of compounds, including cationic, anionic, and neutral substances, such as drugs, metals, and fatty acids (Domenech et al., 2005).

SPC is an aminocyclitol antibiotic that robustly inhibits bacterial protein synthesis by binding to the 30S subunit of the ribosome. However, its effectiveness against mycobacteria is restricted due to inherent resistance mechanisms (Hurst-Hess K. R. et al., 2023). *Ma*b exhibits a notable inherent resistance to SPC, with MIC exceeding 1,000 μg/mL, rendering it unsuitable for therapeutic applications (Hurst-Hess K. R. et al., 2023). The *whiB7* is responsible for *Mab* resistance for SPC because sublethal exposure to SPC strongly induces *whiB7* and its regulon, and a *ΔMab_whiB7* strain shows SPC sensitive (Hurst-Hess K. R. et al., 2023). Furthermore, MAB_2780c, a TetV-like efflux pump, provides high-level SPC resistance in *Mab* (Hurst-Hess K. et al., 2023). For instance, the elimination of *MAB_2780c* resulted in a significant enhancement of susceptibility to SPC, approximately 150 times greater than that observed in the wildtype bacteria (Hurst-Hess K. R. et al., 2023). The inclusion of the efflux pump inhibitor (EPI), verapamil, leads to a reduction in the MIC of SPC by over 100-fold in bacteria that express *MAB_2780c*, bringing it down to levels comparable to those observed for the deletion mutant of *MAB_2780c* (Hurst-Hess K. R. et al., 2023).

#### 2.6.1 Transcriptional regulator TetR and EPs in *Mab*

Recent studies have attributed the drug resistance function of the *Mab* MmpL family. The MmpS-MmpL protein complex provides significant resistance to thiacetazone (TAC) analogs, bedaquiline (BDQ), and CFZ (Gutiérrez et al., 2019). Point mutations occurring in the *MAB_4384* gene of *Mab*, a transcriptional repressor belonging to the TetR family, have been linked to resistance to various drugs. These mutations lead to elevated expression levels of the *MAB_4383c* (*mmpS5*) and *MAB_4382c* (*mmpL5*) genes, ultimately resulting in drug resistance, including resistance to TAC analogs (Halloum et al., 2017; Richard et al., 2018a). In a separate study, Li et al. identified mutations in *MAB_4384* in clinically isolated strains resistant to BDQ (Li et al., 2018). Additionally, Negatu et al. reported an EP’s involvement in LZ resistance. They induced high-level LZ resistance in the *Mab* reference strain *in vitro* and identified resistance mutations in *MAB_4384*, resulting in a lower level of antibiotics resistance for drugs such as Sutezolid (STZ), Tedizolid (TDZ), and TBI-223 (Negatu et al., 2023). This *MAB_4384*-associated lower level of antibiotic resistance was also observed with SPR719 and TAC analogs as well (Halloum et al., 2017; Richard et al., 2018a; Aragaw et al., 2022). The repression of transcriptional expression of two MmpS-MmpL EPs is also facilitated by *MAB_2299c*, which mediates the production of MmpT5 (Alexander et al., 2017). MmpT5 is a member of the TetR family that regulates the expression of the adjacent *mmpS-mmpL* (*MAB_2300–2,301*) genes (Alexander et al., 2017). Mutations in the DNA-binding domain of *MAB_2299c* lead to upregulated EPs, increased drug efflux, and resistance to CFZ and BDQ (Alexander et al., 2017; Richard et al., 2018b). Furthermore, Gutiérrez et al. identified a new target of *MAB_2299c* named *mmpS-mmpL* (*MAB_1135c-MAB_1134c*), which encodes a new MmpS-MmpL EP system involved in intrinsic resistance to CFZ and BDQ (Gutiérrez et al., 2019).

#### 2.6.2 Other EPs in *Mab*

Apart from the above-mentioned EPs, various EP-related genes exist in *Mab*. Two EP-encoding genes, *MAB_1409* and *MAB_3142*, were consistently overexpressed upon exposure to CLR (Vianna et al., 2019). Guo et al. also identified six clinical isolates with CLR resistance among 194 whole-genome sequenced isolates. These resistant isolates, which lacked the common *rrl* 2270/2271 mutation and showed no mutations in the *rrl, rplC, rplD, rplV*, or *erm(41)* genes, exhibited elevated expression of EP genes, specifically *MAB_2355c, MAB_1409c*, and *MAB_1846* (Guo et al., 2020). Additionally, Gorzynski et al. reported the upregulation of *MAB_0937c, MAB_1137c, MAB_4117c*, and *MAB_4237c*, all of which encode EPs and transporter systems when exposed to AMK (Gorzynski et al., 2021). A promising strategy to enhance drug susceptibility involves inhibiting EP activity using EPIs. EPIs are compounds designed to act on EPs and block their efflux function (Song and Wu, 2016; Remm et al., 2022). Several experimental examples demonstrate the use of EPIs to increase the susceptibility of anti-*Mab* agents. In a study by Vianna et al., the crucial role of efflux activity in *Mab* resistance to CLR was highlighted. This was evident in the increased mRNA expression levels of *MAB_1409* and *MAB_3142* in *Mab* after exposure to CLR. Moreover, the researchers discovered that verapamil (VP), an FDA-approved EPI with potential as adjunctive chemotherapy for tuberculosis, significantly enhanced susceptibility to CLR. This effect was observed across *Mab* clinical isolates belonging to the T28 *erm(41)* sequevar., known for inducible resistance to CLR (Vianna et al., 2019). In addition, Guo et al. reported that the presence of EPIs, such as phenylalanine-arginine β-naphthylamide (PAβN), a peptidomimetic compound, carbonyl cyanide m-chlorophenylhydrazone (CCCP), and VP, significantly decreased the MIC of CLR for *Mab* resistant isolates that exhibited no *rrl* 2270/2271 mutation (Guo et al., 2020).

### 2.7 Genetic polymorphism of target genes

Genetic polymorphisms in highly conserved genes targeted by pharmaceutical medications have been associated with variations in sensitivity to pharmacological effects in *Mab* infections (Nessar et al., 2012).

#### 2.7.1 Ethambutol

Ethambutol and fluoroquinolone resistance serve as examples of genetic polymorphism influencing drug resistance. Ethambutol is chemically derived from ethylenediamine, possessing the stereochemical structure S,S, achieved by substituting a hydrogen atom on each nitrogen atom of ethane-1,2-diamine with a 1-hydroxybutan-2-yl group (Jahangir et al., 2016). Ethambutol functions as a bacteriostatic medication against mycobacterial infections by inhibiting cell wall formation in these microorganisms (Sreevatsan et al., 1997). However, *Mab* exhibits substantial inherent resistance to ethambutol, primarily due to variant nucleotides within the conserved ethambutol resistance-determining region (ERDR) of the *embB* gene (Alcaide et al., 1997). *Mab* shows amino acid substitutions, particularly the replacement of isoleucine by glutamine at position 303 and leucine by methionine at position 304 (referred to as I303Q and L304M, respectively) (Sreevatsan et al., 1997).

#### 2.7.2 Fluoroquinolone

Another example of genetic polymorphism influencing drug resistance involves fluoroquinolones. These antibiotics encompass over 20 medications originating from the identification of nalidixic acid. Derived from the quinolone family, fluoroquinolones are synthetic compounds formed by modifying 1-alkyl-1,8-naphthyridin-4-one-3-carboxylic acid. Fluoroquinolones strongly inhibit bacterial enzymes, DNA gyrase, and topoisomerase, critical for processes like DNA replication (Redgrave et al., 2014). They are secondary therapeutic agents for multi-drug resistant tuberculosis (MDR-TB), working by inhibiting DNA gyrase’s supercoiling activity, a specific target of fluoroquinolones (Pantel et al., 2011). NTM resistance to fluoroquinolones is predominantly due to genetic factors, particularly the analysis of conserved sections called quinolone resistance-determining regions (QRDRs) within DNA gyrase subunits GyrA and GyrB, which are the primary targets of quinolone drugs. In *Mab*, resistance results from the presence of alanine at position 83 (Ala-83) in the GyrA QRDR. Furthermore, resistance is conferred by arginine at position 447 (Arg-447) and asparagine at position 464 (Asn-464) within the GyrB QRDR (Matrat et al., 2008).

#### 2.7.3 Telacebec (Q203)

Telacebec (Q203) is a groundbreaking anti-tuberculosis drug designed to inhibit the cytochrome *bc_1_* complex, affecting cellular energy production in *Mtb*. This inhibition reduces ATP synthesis, halting bacterial growth (Pethe et al., 2013; de Jager et al., 2020). Notably, Q203 lacks inhibitory activity against *Mab*. A recent study by Sorayah et al. revealed that naturally occurring polymorphisms within *Mab* QcrB are responsible for its increased resistance to Q203. To confirm this resistance mechanism, they engineered a *Mycobacterium bovis* BCG strain, integrating the chimeric *Mab qcrCAB* operon, where four amino acids (D311E, L314A, G179S, and C393A on QcrB) were modified to match their counterparts in *Mtb*. This genetic adjustment rendered the chimeric *M. bovis* BCG strain susceptible to Q203, indicating that *Mab*’s resistance to Q203 is attributable to naturally occurring polymorphisms in the drug target, QcrB, rather than other inherent resistance mechanisms such as efflux pumps, cell wall permeability, or target-modifying enzymes (Sorayah et al., 2019).

## 3 Acquired drug resistance of newly developing compounds against *Mab*

### 3.1 Epetraborole

In recent years, aminoacyl-tRNA synthetases (AARSs) have become significant targets for new antibacterial interventions. AARSs facilitate the acylation process, linking amino acids and tRNA. Inhibiting AARS activity halts protein synthesis, impeding bacterial growth. Benzoxaboroles, known as boron-heterocyclic antibiotics, inhibit leucyl-tRNA synthetase (LeuRS) (Rock et al., 2007). These drugs obstruct protein synthesis via the oxaborole tRNA-trapping mechanism, forming adducts with uncharged tRNALeu molecules that bind to the LeuRS editing domain (Rock et al., 2007). Multiple reports discuss the anti-*Mab* effects of LeuRS inhibitors, particularly a new class of LeuRS inhibitors such as epetraborole, DS86760016, EC/11770, MRX-6038, and GSK656 (Kim et al., 2021; Wu et al., 2022; Ganapathy et al., 2023a). High-level epetraborole resistance may be attributed to mutations in *Mab* at locations S303L, T322I, T323P, F321V, G393V, and Y421D within the LeuS gene (Ganapathy et al., 2021a). Among the LeuRS inhibitors, DS86760016 exhibits an improved pharmacokinetic profile, lower plasma clearance, longer plasma half-life, and higher renal excretion than epetraborole in animal models. A recent study by Nguyen et al. demonstrated that DS86760016 displayed similar activity to epetraborole treatment against *Mab in vitro*, intracellularly, and in zebrafish infection models, with a significantly lower mutation frequency. Laboratory-induced DS86760016-resistant strains included D284G, Q345R, Y420C, I426T, V468L, N469Y, and E524K, which were not found on the LeuS gene in epetraborole-resistant mutants (Nguyen et al., 2023).

### 3.2 MmpL3 inhibitor

Mycolic acids (MA) exist in various forms, including trehalose monomycolates (TMMs), trehalose dimycolates (TDMs), and mycolates covalently linked to arabinogalactan (AG) polysaccharides (McNeil et al., 2020). In the process of MA synthesis, MmpL3 plays a crucial role by transporting MA across the inner membrane, making its contribution to cell wall production indispensable (McNeil et al., 2020). Inhibition of the MmpL3 transporter leads to the accumulation of TMM intracellularly, causing a decrease in mycolyl arabinogalactan peptidoglycan (mAGP) and TDM levels (McNeil et al., 2020). Consequently, MmpL3 represents a versatile drug target, and inhibiting MmpL3 disrupts cell wall biosynthesis (Li et al., 2014). Multiple MmpL3 inhibitors have been recently identified through phenotypic screenings of chemical libraries against *Mab*. Promisingly, PIPD1, a piperidinol-based molecule, has shown potent *in vitro* and *in vivo* activity against clinical *Mab* strains. Treatment of infected zebrafish with PIPD1 increased embryo survival and reduced bacterial burden (Dupont et al., 2016). Major resistance to PIPD1 and other MmpL3 inhibitors, such as EJMCh-6 (2-(2-cyclohexylethyl)-5,6-dimethyl-1H-benzo[d]imidazole) and BMC-2i, is attributed to mutations in the MmpL3 gene (*MAB_4508*) of *Mab* at location A309P. The overexpression of MmpL3, containing the Ala309Pro mutation in *Mab* wild-type bacteria, results in significant drug resistance to MmpL3 inhibitors, confirming MmpL3 as their target (Dupont et al., 2016; Kozikowski et al., 2017).

### 3.3 Delpazolid (DPZ)

LZ, a representative oxazolidinone, disrupts protein synthesis by inhibiting the peptidyl transferase activity of the 23S rRNA in the 50S ribosomal subunit, leading to ribosome stalling. Unfortunately, most clinical *Mab* isolates exhibit poor susceptibility to LZ (Negatu et al., 2023). However, the effectiveness of LZ against NTMs varies among different derivatives, and its clinical use in patients with NTMs can sometimes result in adverse events, such as peripheral neuropathy and cytopenias. Recently, Kim et al. introduced a novel oxazolidinone with a cyclic amidrazone named DPZ (LCB01-0371). DPZ demonstrated effective inhibition of *Mab* growth, both *in vitro* and in mouse lungs *in vivo* compared to LZ. Furthermore, DPZ exhibited bactericidal activity against all bacterial strains, irrespective of their resistance to AMK, CFX, or CLR. Kim et al. generated laboratory-induced resistant mutants to DPZ and identified mutations in *rplC* (encoding 50S ribosomal protein L3) at T424C and G419A, along with a nucleotide insertion at position 503 through sequencing analysis (Kim et al., 2017).

### 3.4 SPR719

The aminobenzimidazole SPR719 targets the ATPase located on Gyrase B in *Mtb*. SPR719 also demonstrates activity against NTM and has recently entered clinical trials for lung diseases caused by NTM (Aragaw et al., 2022). Brown-Elliott et al. (2018) demonstrated *in vitro* activity of SPR719 against *M. abscessus, M. massiliense*, and related subspecies, with an observed MIC_50_ (MIC required to inhibit the growth of 50% of *Mab*) value of ~2.0 μg/mL. Additionally, Rubio et al. reported that the phosphate prodrug SPR720 (of SPR719) exhibited favorable *in vivo* efficacy at a dose of 100 mg/kg/day in an SCID mouse model infected with *Mab* (Egorova et al., 2021). To identify the molecular target for *Mab*, Aragaw et al. recently induced two different morphotypes of SPR719-resistant mutants on agar containing 16 x MIC of SPR719, named large and small colonies. Interestingly, the small colony phenotype reflected a lower level of resistance, while large colonies showed high-level SPR719 resistance (>16-fold MIC increase). All strains contained a single amino acid polymorphism, Thr169Asn, in the ATPase domain of Gyrase B, and non-*gyrB* DNA sequence polymorphisms were revealed by whole-genome sequencing. Thr169 in *Mab* DNA gyrase corresponds to Ser169 in *Mtb* GyrB, causing resistance to SPR719 in *Mtb* (Aragaw et al., 2022).

## 4 Concluding remarks

*Mab* has emerged as a significant threat to human health, posing challenges in treatment due to its resistance to currently available commercial medications (Quang and Jang, 2021). The rising number of publications on NTM, especially *Mab*, signifies exponential growth. However, the level of attention dedicated to this issue remains insufficient to effectively address the problem. The primary challenge in developing drugs for *Mab* is attributed to its exceptional innate and acquired resistance capabilities (Wu et al., 2018). High drug resistance discourages investments by both pharmaceutical companies and governments in this area, leading to passive involvement. As a result, small- and medium-sized organizations, along with academic institutions, are currently the primary sources of knowledge regarding *Mab* infections and antibiotics. Antibiotic resistance in mycobacterial species can occur through natural or acquired mechanisms (Nessar et al., 2012). Development of natural drug resistance in *Mab* can be attributed to several factors, including a waxy impermeable cell wall acting as both a physical and chemical barrier (Nessar et al., 2012). Additionally, drug export systems, enzymes capable of modifying drugs or target enzymes, and genetic polymorphism in target genes contribute to this phenomenon (Nessar et al., 2012). Acquired resistance arises from spontaneous mutations at specific genes in response to antibiotics following extended treatment (Johansen et al., 2020b). Such mutations alter the target gene or other related genes, rendering the medication ineffective (Johansen et al., 2020b). Our understanding of natural or acquired antibiotic resistance in *Mab* remains limited, highlighting the importance of further research into resistance mechanisms against current antibiotics and the discovery of new compounds to overcome resistance hurdles and develop novel drugs.

## Author contributions

TN: Investigation, Methodology, Validation, Writing – original draft. BH: Investigation, Methodology, Validation, Writing – review & editing. SJ: Investigation, Methodology, Validation, Writing – review & editing. AA: Investigation, Methodology, Validation, Writing – review & editing. HL: Investigation, Methodology, Validation, Writing – review & editing. CM: Investigation, Methodology, Validation, Writing – review & editing. JJ: Conceptualization, Funding acquisition, Methodology, Validation, Writing – original draft, Writing – review & editing.
