# Supplementary material for: Exploring antibiotic resistance mechanisms in Mycobacterium abscessus for enhanced therapeutic approaches
Source: Front Microbiol. 2024 Feb 6;15:1331508. doi: 10.3389/fmicb.2024.1331508 (PMC10877060; doi:10.3389/fmicb.2024.1331508)
Supplement: Supplementary file 1 [file Presentation_1.PPTX]

## Slide 1
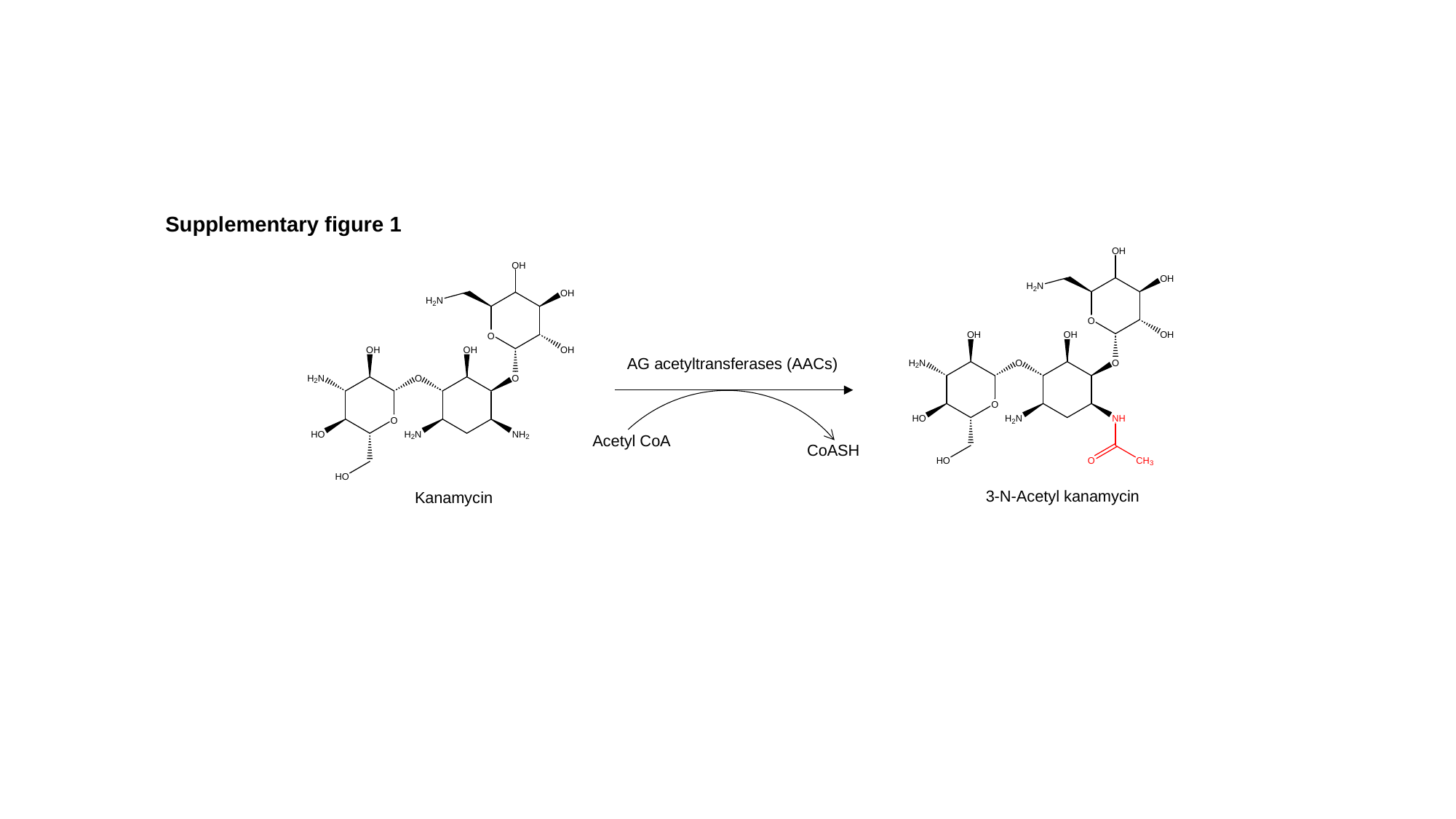

Supplementary figure 1
AG acetyltransferases (AACs)
Acetyl CoA
CoASH
3-N-Acetyl kanamycin
Kanamycin

## Slide 2
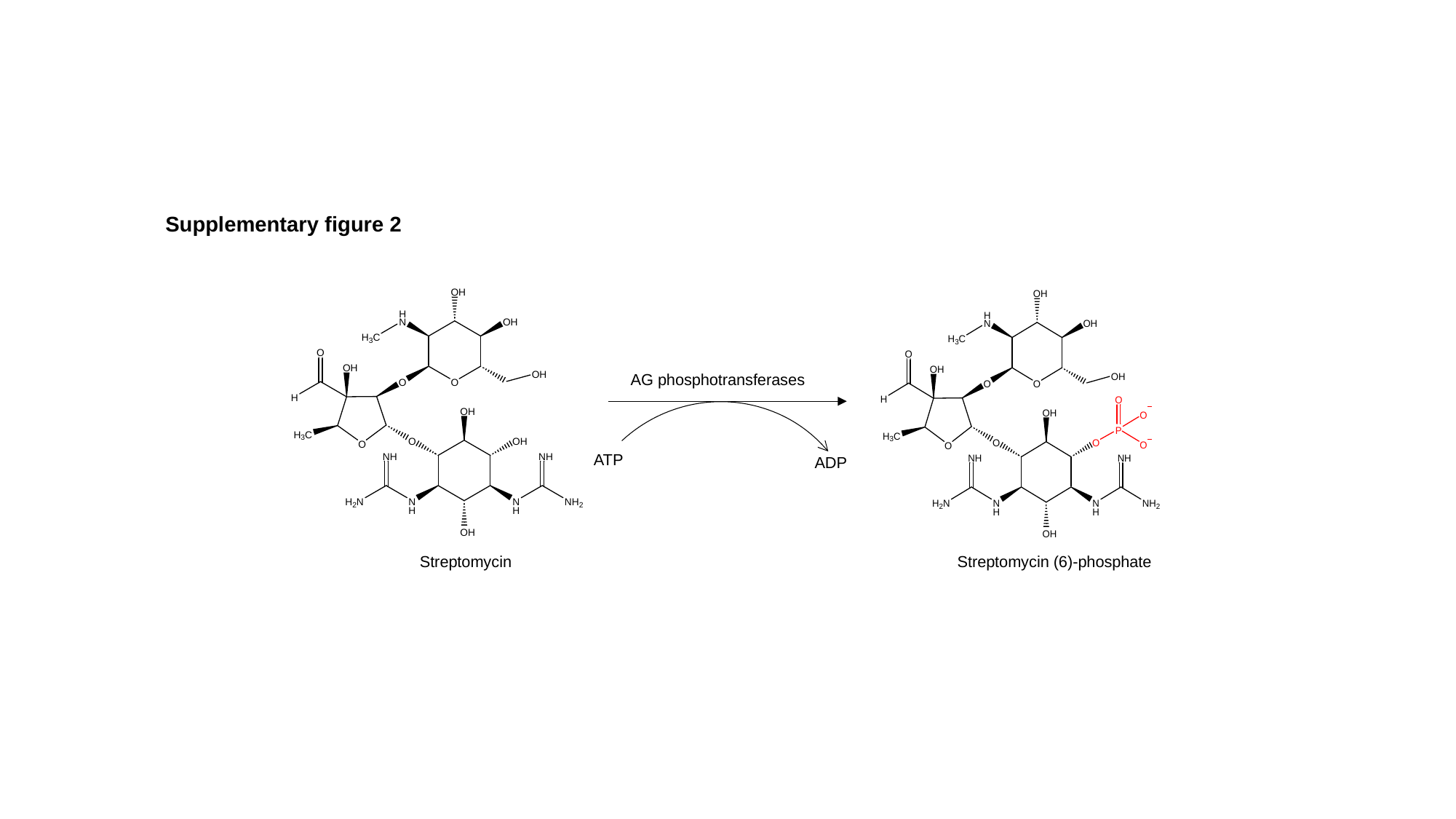

Supplementary figure 2
AG phosphotransferases
ATP
ADP
Streptomycin
Streptomycin (6)-phosphate

## Slide 3
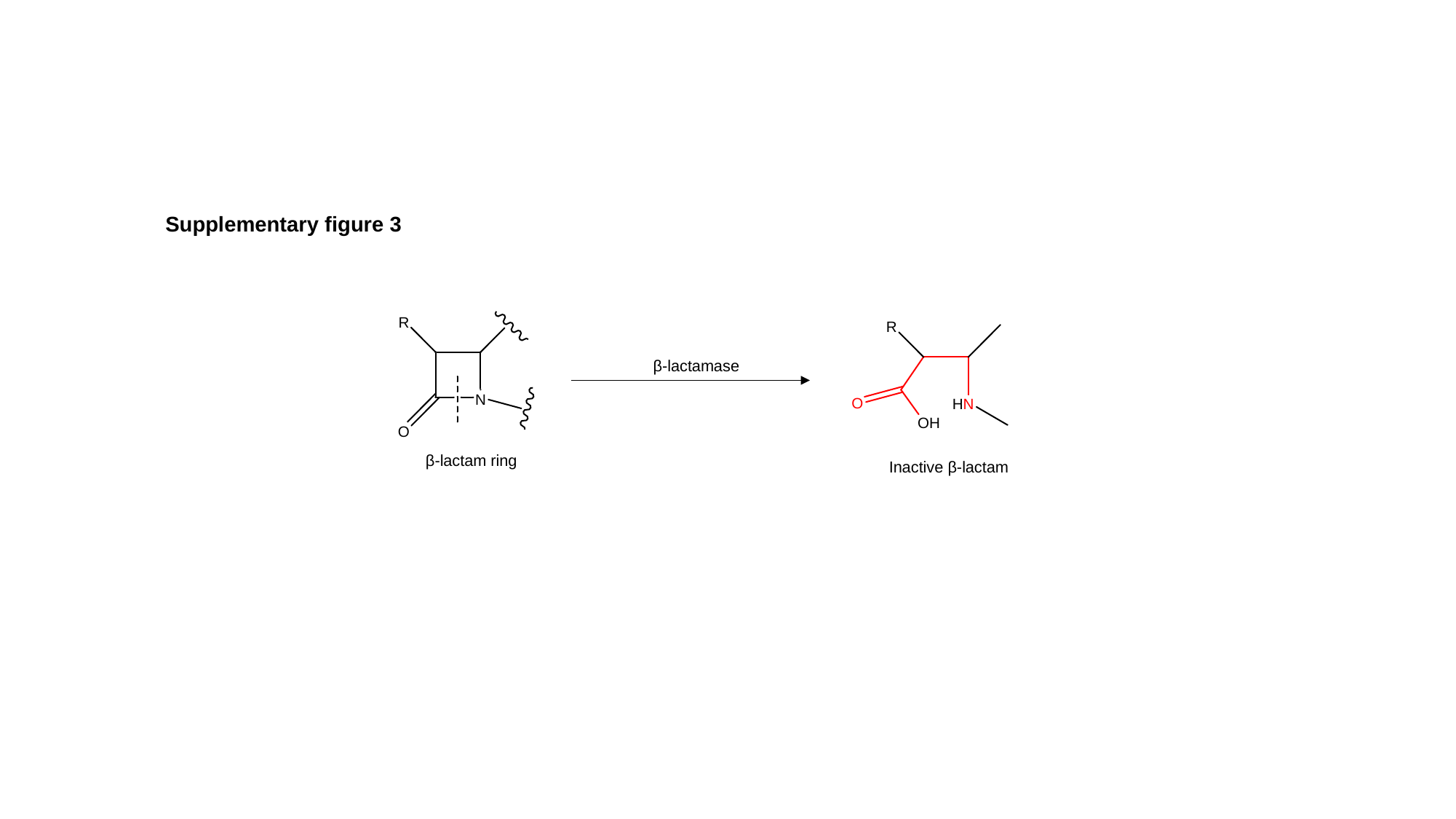

Supplementary figure 3
β-lactamase
β-lactam ring
Inactive β-lactam

## Slide 4
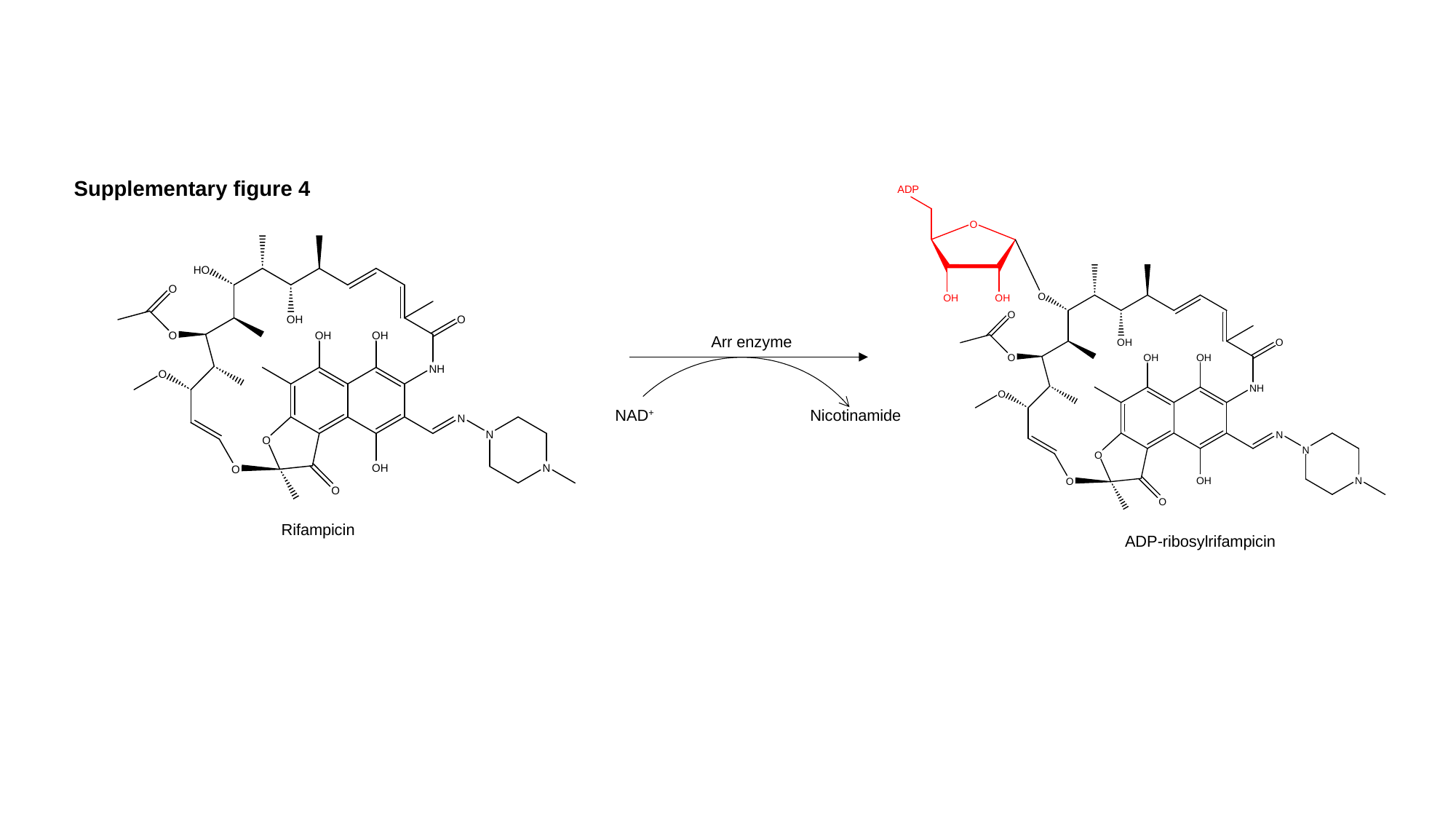

Supplementary figure 4
ADP
Arr enzyme
NAD+
Nicotinamide
Rifampicin
ADP-ribosylrifampicin
